# Non-medical aspects of civilian–military collaboration in management of major incidents

**DOI:** 10.1007/s00068-017-0778-6

**Published:** 2017-03-02

**Authors:** A. Khorram-Manesh, H. Lönroth, P. Rotter, M. Wilhelmsson, J. Aremyr, A. Berner, A. Nero Andersson, E. Carlström

**Affiliations:** 1 Prehospital and Disaster Medicine Center, Regionens Hus, 405 44 Gothenburg, Sweden; 2 Department of Surgery, Institute of Clinical Sciences, Sahlgrenska Academy, Gothenburg University, Gothenburg, Sweden; 3 Department of Health and Crisis Management and Policy, Sahlgrenska Academy, Gothenburg University, Gothenburg, Sweden

**Keywords:** Civilian–military, Trauma, Terrorism, Collaboration, Simulation, Exercise

## Abstract

**Purpose:**

Disasters and major incidents demand a multidisciplinary management. Recent experiences from terrorist attacks worldwide have resulted in a search for better assessment of the needs, resources, and knowledge in the medical and non-medical management of these incidents and also actualized the need for collaboration between civilian and military healthcare. The aim of this study was to evaluate the impact of the civilian–military collaboration in a Swedish context with the main focus on its non-medical management.

**Method:**

An exercise, simulating a foreign military attack centrally on Swedish soil, was designed, initiated, and conducted by a team consisting of civilian and military staff. Data were collected prospectively and evaluated by an expert team.

**Results:**

Specific practical and technical issues were presented in collaboration between civilian and military staffs. In addition, shortcomings in decision-making, follow-up, communication, and collaboration due to prominent lack of training and exercising the tasks and positions in all managerial levels of the hospital were identified.

**Conclusion:**

Current social and political unrests and terror attacks worldwide necessitate civilian–military collaboration. Such collaboration, however, needs to be synchronized and adjusted to avoid preventable medical and non-medical consequences. Simulation exercises might be one important source to improve such collaboration.

## Introduction

The increasing risk of natural disasters, as well as, major incidents created by human, such as terror attacks, indicates a need for collaboration between different agencies [1, 2]. In 2010, during the devastating flooding, which killed 2000 people and affected 20 million people, the Pakistani military troops were mobilized to collaborate and assist the civilian agencies, e.g., healthcare. They accomplished immediate life-saving assistance, including rations from the military’s supplies as well as field hospitals and relief camps. Other examples of civilian–military (CM) collaboration are Thailand during the Indian Ocean tsunami 2004, the 2005 Pakistan earthquake, and the 2010 Haiti earthquake [1].

Recent experiences from terrorist attacks worldwide, particularly in Europe (France 2015), have resulted in a search for better assessment of the needs, resources, and knowledge in the medical and non-medical management of these incidents [2, 6]. As the attacks vary in type, magnitude, and outcomes, and result in injuries, which are rarely treated in a civilian setting, the needs for a wider planning and CM healthcare collaboration is obvious. The CM collaboration in the medical management of victims is often high lightened and mentioned as one of the major cause of a successful outcome [2–7].

Although CM collaboration is common in battle areas, especially within the field of healthcare, relatively few studies have scrutinized the effects of such collaboration from a civilian perspective [4, 5]. In all examples of overwhelming mass casualties, the civilian society is in dire need of support from military resources. However, the opposite situation may exist, i.e., the military might be in need of civilian healthcare resources during a military action. An assumption in such situation would be that such collaboration puts the civilian medical community and trauma centers into a test. The challenges would be both medical and non-medical. The medical challenges would be: the knowledge of damage control management at prehospital and hospital levels; the ability to treat immediate survivors with military injury patterns; the outcome of such treatment in terms of mortality and morbidity; etc [2, 5–10]. The non-medical issues, such as command and control, communication, collaboration, logistic, information etc, are fairly well discussed in the literature, but few studies have prospectively analyzed the impact of a CM collaboration in respect to these topics [3, 4, 6]. Some of the issues might be solved based on the facts and knowledge, and some by relying on the experience and background of involved managers at operational and tactical levels [11].

To analyze the non-medical outcomes of a CM collaboration, the focus should mainly be on the organizational and logistic interface of the CM healthcare. Although there are many guidelines, which regulate CM collaboration, they are not adopted to conflicts in western countries. The Oslo guidelines developed in 1994 and updated in 2006 address only natural disasters in times of peace. The MDCA guidelines (guidelines on the use of military and civilian assets to support UN humanitarian activities in complex emergencies) from 2003 are developed to suit humanitarian actions in countries, such as Iraq and Afghanistan, where the foreign military has been in the presence [12].

The aim of this paper was to analyze the outcomes of CM collaboration in a mutual exercise, in which military casualties were brought to a civilian hospital for further care. The focus was mainly on the surge capacity and organizational structure, command and control, participants knowledge about their responsibilities, functions, and organizational belonging during a major incident, their ability of understanding the nature of issues that may appear during an incident, such as triage of resources, organizational maintenance, safety and communication, logistic, and the need and demands for developing CM collaboration.

The scenario used in this exercise was a foreign military attack centrally on the western region of Sweden. A prominent industrial zone in Sweden with 1.6 million inhabitants, living in urban as well as rural, and scarcely populated areas with five major hospitals with emergency departments (ED) and emergency care competency [13]. Complementary care in some specialties (e.g., Neurosurgery, vascular surgery, etc) is provided by the university hospital in Gothenburg; the only trauma center in the region. The attack generated a varying number of casualties (*n* = 28) with typical war injuries. They were transported by military helicopters, either as single victims or two on the same helicopter, from the field hospital/s to the trauma center in Gothenburg. They were all landed on the helipad and delivered to the civilian staff after a report exchange and were admitted through the ED on Monday 31 August 2015, starting at 08:00 a.m.

## Method and material

### Evaluation template and observations

Data were collected prospectively using an evaluation template based on CSCATTT [14]. This template consisted of the following sections: Command and control, Safety, Communication, Assessment, Triage, Treatment, and Transport were reviewed and evaluated by free text. Observers were allowed to comment all activities, pros, and cons and if possible, and evaluate any specific activity by giving a point between 1 and 10 on a VAS scale (10 = excellent).

During the observations, all observers accompanied and observed the military and hospital employees at different stations (see in the following) [15]. The raw data could be collected, even though the event itself proceeded relatively fast [16]. The analysis was carried out in three steps. First, we selected the relevant effects of military–civil integration within the exercise. In the second step, we coded the findings. Then, we sorted and analyzed all the observations as a whole [17].

A total number of nine observers/evaluators were engaged in the evaluation of scenario and were localized at the following station;

*Helipad* The place for first communication between military and civilian staff.

*Transportation path* From helipad to ED

*ED* The first place to examine, triage, and manage all victims at admission and trauma bay

*Hospital command group (HCG)* A group of decision makers, with local responsibilities to organize, assess, and maintain the local needs at the hospital.

The total time of observations was 63 h.

## Results

The results indicating the shortcomings and the points for improving are given for each station. Since some of the issues may be related to the shortcomings in hospitals contingency plan and not CM collaboration, all issues were divided into issues related to CM collaboration (A) and those related to hospitals contingency plan (B).

### Helipad

#### Issues related to CM collaboration

##### Reporting system

Different reporting systems between military and civilian healthcare staff were identified. The military staff used ATMIST (Age of the patient, Time of the incident, Mechanism of injury, Injury, Signs, Treatment) or MIST (Mechanism of injury, Injury, Signs, Treatment), while civilian staff used SBAR (Situation, Background, Actual, Recommendations). This discrepancy in reporting resulted in misunderstanding, low situation awareness, missing vital patient information, and initiation of unnecessary measures. Five important points in the process of reporting (identifying right person to report to, silent minute to absorb the information and let the report flow, content of the report, standardization of the report and documentation) were particularly noted by observer/evaluator in ten randomly chosen red patients and were given a point on the VAS scale, as mentioned in “Method” (Table 1). The lack of standardization in the reports and a proper documentation were prominent.

**Table 1:** Military-to-civilian reporting process on the helipad in ten randomly chosen red patients

Patient	A	B	C	D	E	F	G	H	I	J	Average
Identifying the right person to report	2	6	8	8	8	8	10	8	10	10	7.6
Silence minute	2	6	10	10	10	10	10	10	10	10	8.8
Content of the report	4	6	8	8	8	8	10	8	8	10	7.8
Standardized report	2	4	4	4	4	4	8	4	4	8	4.2
Documentation	4	4	6	6	6	6	6	6	4	6	5.4

10 = excellent

##### Protocol

**Fig. 1:**
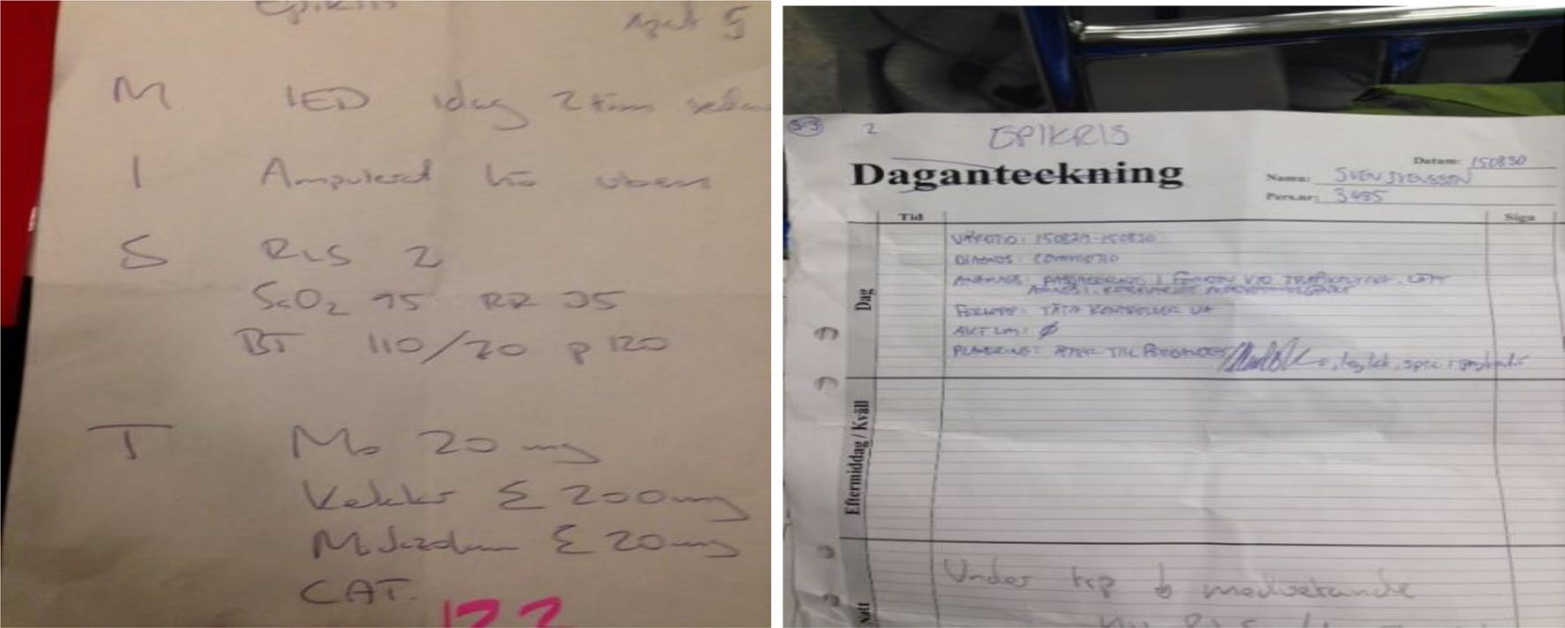
Readability of the hand-written report

There was no standardized protocol for reporting the patient’s medical history and/or medical signs between military and civilian staffs. This resulted in a hand-written report, which was time-consuming, hard to read and lacked vital information (Fig. 1).

##### Noise

In comparison with a civilian helicopter, a military helicopter never turns off its motor as practiced during the war. Consequently, the whole report was given under functioning helicopter and created a noisier environment in which key information was lost.

##### Military stretchers

Patients were carried on military stretchers, which were supposed to be changed at the time of patient delivery with a similar one at the hospital. However, the military and civilian stretchers were not compatible, and therefore, the military stretchers had to be returned. The process of off-loading patients from military stretchers and re-loading to a civilian stretcher was time-consuming and jeopardized the patient safety.

#### Issues related to contingency plan

##### Wind

During the exercise, it was very difficult to hold loose items, including triage tags, medical items, papers, etc, due to the winds and the turbulence from the helicopter. There was no covering area, which could protect patients and staff from rain, wind, and cold.

### Hospital logistic; transport path to the ED

#### Related to CM collaboration

##### Medical resources

Due to the high number of casualties and consequent multiple helicopter landings, staff and instrument were not in place all the time. With each nurse leaving the scene, her replacement could delay, since they used the same path. In a daily and customary basis, there would be few helicopter arrivals, which can easily be handled.

##### Reporting times

The time for giving report was extremely short at the trauma bay (15 s) compared to report given on helipad (45 s). There were obvious problems with the presentation of the hand-written report from the helipad area to the trauma team (Table 2). Looking at the same parameters in the process of reporting (identifying right person to report to, silent minute to absorb the information and let the report flow, content of the report, and standardization of the report and documentation); beside the lack of standardization and documentation, the scarce content of the report was also prominent.

**Table 2:** Reporting time consumed in civilian–civilian reporting process for the patients in this study

Patient	A	B	C	D	E	F	G	H	I	J	Average
Identifying the person who receives the report	10	10	10	10	10	10	10	10	10	10	10
Silence minute to let report flow	10	10	10	10	10	10	10	10	10	10	10
Content of the report	4	4	4	8	6	8	8	1	6	6	5.5
Standardized report	0	0	0	0	0	0	0	0	4	0	0.4
Documentation	6	2	6	6	4	6	4	2	4	4	4.4

10 = excellent

### Related to contingency plan

#### Transport time to the ED

Table 3 shows the time from helicopter landing to the time when the patient was reported to the trauma room. The transport time varied between 9 and 16 (average 11.9) min. Delays occurred if elevators were not running fast. The time was much longer if two patients were delivered by the military helicopter. The table shows the time consumed for ten randomly chosen patients at different levels from landing to the end of reporting at trauma bay. The last column shows the average of consumed time in each level.

**Table 3:** Time from helicopter landing until the patient is delivered to the ED

Patient	A	B	C	D	E	F	G	H	I	J	Average
Helicopter lands	0	0	0	0	0	0	0	0	0	0	0
Start reporting on helipad	+4	+4	+5	+2	+3	+4	+5	+3	+3	+4	3.7
End reporting on helipad	+1	+0	+4	+1	+0	+1	+1	+1	+1	+0	1.0
At the elevator (helipad)	+3	+2	+2	+1	+1	+3	+1	+1	+1	+1	1.6
Arrival at ED	+4	+4	+4	+4	+4	+5	+5	+4	+4	+5	4.3
Start reporting at trauma bay	+1	+1	+1	+1	+1	+1	+1	+2	+1	+1	1.1
End reporting at trauma bay	+0	+0	+0	+1	+0	+0	+0	+0	+1	+0	0.2
Total time (min)	13	11	16	10	9	14	13	11	11	11	11.9

#### Competency during patient transportation

Almost all patients were transported by a single nurse together with another caretaker with no medical knowledge. Any deviation in patients’ vital signs could not be medically responded easily with only one nurse available.

#### Communication

The phone used by the nurse stopped working in periods during transportation and mainly in the elevator, thus leaving the sole nurse alone should anything severe happen. In eight out of ten victims, triaged as severe cases (red tags), there was a need for additional qualified staff during transportation to maintain the high quality and ensure the safety of patients during transportation.

### ED (emergency department)

#### Related to CM collaboration

None.

#### Related to contingency plan

##### Teamwork and organization

Good situation awareness, initially common understanding of the incident, review of the mass casualty plan, and good preparation. However, with the arrival of victims, ED was transferred to a more disorganized area, stepwise, e.g., some of the staff could not find the way to the helipad. Traffic jam at the entrance and exit of the trauma bay area. There were two doors, but just one was used.

##### Information

There was a misunderstanding about the number of casualties in many occasions, especially after a while when the number of receiving patients did not match the number of existing patients. The information given to the staff at trauma bay was scarce and many wondered where the patient come from? No one realized that the victims came from field hospitals. There was a lack of information about, where the victims went to and with which medical files? The exercise was over, however, no one knew who made the decision, when and why?

##### Communication

Wrong numbers on the list delay the arrival of the surgeon and anesthesiologist. No connection to the emergency laboratory probably due to the network problem. Communication problem between staff on the helipad, ED, and intensive care unit concerning where to patients should be transported.

##### Documentation

Only one secretary worked with documentation at trauma bay and she was overloaded. There was also a confusion about which admission template should be used, the ordinary one or the one designated for disaster?

##### Resources

Neck stabilizing collars in the trauma bay were not enough.

The performance of ED at two levels, outside and inside trauma bay, based on a preplanned template with specific topics, is given in detail in Table 4. Points were given based on the VAS scale (Table 4).

**Table 4:** Evaluation of ED’s organizational structure in VAS scale

	Points	Comments
Outside trauma bay		
Obtaining information	5	
Analyzing ability	5	
Decision-making ability	8	No follow-up and analysis
Follow-up a decision	6	See above
Team/organizational build-up	9	Initially good, but worsen with chaos
Establishing contact with commanders	9	Excellent
Working areas	5	Not good for commanding
Resources	6	Scarce
Individual equipment	8	Some lacking
Information sharing	5	Unclear sometimes
Cooperation between physicians	9	
Identifying receiver of the report	6	Who needs the information and how?
Silence minute	1	None
Standardized reporting template	1	None
Written information	5	Not complete e.g. alerting level
Assessment of the situation	5	Number of patients vs available beds
Communication with other units	7	
Inside trauma bay		
Team/organization build-up	7	
Admission of patients	4	
Working area	7	Long distance to helipad and intensive care unit
Available resources	8	Not realistic, hard to measure
Management of patients	8	ATLS-based
Identifying receiver of information	8	
Silence minute	7	Most of the time
Contents of the report	5	Large variation
Report template	3	None
Situation assessment	9	
ATLS guidelines	9	Yes
Patients transported to known space	9	Yes
Teamwork	9	Yes
Equipment	9	Yes

Ten points = excellent

### Hospital command group (HCG)

#### Related to CM collaboration

None.

#### Related to contingency plan

##### Teamwork and organization

Smooth teamwork early in the process, in designated room, based on the hospitals´ disaster and contingency plan, in a seemingly well-equipped room.

##### Communication

Problem, especially concerning the level of preparedness.

##### Information

They had a timely press release. However, it was unclear if the amount and content of released information were known to the public, emergency medical services, others emergency departments, primary care, as well as internally?

##### Follow-up

Decision made or scenarios discussed were not followed up.

##### Instruments

Problem with conference and communication devices sporadically.

The performance of HCG, based on a preplanned template with specific topics, is given in detail in Table 5. Points were given based on the VAS scale (Table 5).

**Table 5:** Evaluation of HCG in VAS scale points 1–10

Action	Point	Comments
Obtaining information	7	Was received through telephone calls and meetings. However, information obtained was not shared adequately
Analyzing ability	7	High, but not in all functions
Decision-making ability	8	Very high, but not in all functions
Follow-up of decisions made	5	No clear follow-up routine including unclear distribution of tasks. Lack of visual overview of what, who, when and how?
Teamwork/organizational form	7	Good, including participation of all members in discussion, assessments, and suggestions Supporting each other in an overloaded situation Good knowledge about self- and each other’s role and good interaction
Cooperation	7	Good
Coordination	7	Good
Communication	6	Confusing Technically problematic
Identifying report receiver	7	Very good
Reporting template	1	None
Written information	4	Not in all occasions
Situation assessment	9	Excellent

10 = excellent

## Discussion

A successful CM collaboration in the medical management of major incidents relies not only on the skills and knowledge of the staff but also on non-medical compatibility and harmonization of both organizations. Maybe, the strongest message sent by this study is that any kind of multidisciplinary management should be harmonized and tested for compatibility by mutual planning, training and exercising [2, 18–21].

Different countries have different health care systems that may impact the military involvement in civilian disasters and civilian involvement in military conflicts [2]. Some countries have military hospitals, which treat military staff and their families in peacetime and military casualties during the war, and thus, military victims will not be treated in the civilian sector, e.g., USA. Other countries have no military hospitals and civilian hospitals are responsible for the treatment of military casualties, e.g., Israel. In Sweden, there are no military hospitals and emergency care is offered to the staff through full-time or part-time employed military physicians and nurses in collaboration with the university hospitals. If necessary, patients can be referred to the hospitals for further assessment, treatment, and surgical interventions. During an armed conflict or war, Swedish army relies fully on the civilian resources [22].

The collaboration between civilian and military healthcare has long been discussed and recommended mainly due to the civilian healthcare’s economic austerity and its need for sharing resources in terms of manpower and medical devices, vehicles, etc. A new and important reason is the increasing number of terror attacks in last decade with new patterns of injuries (gunshot and explosives) that are totally new to the civilian healthcare [2, 6]. With an increasing number of social disturbances encumbering many countries and putting them in an everyday alertness, any involvement in an armed conflict implicates another burden to their healthcare that must be prepared for [2, 13, 23–27]. Such preparedness should be multidisciplinary planned, tested and conducted [18–20].

It is already known that the medical management of mass casualties needs special consideration and calls for a CM collaboration [2]; however, the non-medical collaboration should also be prepared. In this study, by dividing our findings into CM collaboration-related and contingency plan-related issues, we could identify those issues that particularly should be addressed within the CM collaboration framework.

There was no surprise that most of the issues in the interface of CM collaboration occurred in the area, where they meet for the first encounter, i.e., helipad and during the transportation. Issues such as functioning helicopter during the reporting, non-compatible stretchers, different reporting systems, not only jeopardize patient’s safety but also creates unnecessary and time-consuming measures, which have an impact on the outcome of whole management. The experience gained from recent attacks in France is good enough to realize that a harmonized multidisciplinary approach is a necessity in the management of disasters and major incidents [2, 6]. Standardization is possible by introducing mutual protocols, training, and guidelines [20].

Other issues found in this report were directly related to the hospital and its contingency plan. The long transport path to the ED (256 m of underground culverts, including two elevators) is all structural issues that may jeopardize patient safety. Although the hospital had a valid and comprehensive plan, the people using it were not trained and familiar with the operational items. The lack of training and exercising the tasks and positions was prominent in this study and in different positions (e.g., how to perform the tasks, how to find the way to different sections, how to use various routines, how to identify the right partner for collaboration, and how to analyze and react to unplanned issues). This cannot be done by just a written text in a plan, and a good preparedness is achieved by being exposed to the possible event, new scenarios, exercises, and training [20, 21, 24].

To maintain a high level of preparedness, special considerations should be paid to the preparedness pyramid, which identifies planning, infrastructure, knowledge, and capabilities as the major components [26]. Since all Swedish hospitals have both disaster plans and good infrastructure, the exercise itself gave us a chance to evaluate the level of knowledge and capabilities and thus complete the preparedness pyramid. Studying command and control, communication and collaboration at the hospital, the HCG showed good ability in commanding, group dynamic, and teamwork. However, they were not strong enough in control, communication, and collaboration with other agencies or units. One of the major problems was the inability of following up various decisions or plans, which were made and discussed. Alongside the good discussion and democratic decision-making, it was often forgotten to point out a specific individual to follow a specific task and order [11, 23, 27–29].

Communication internally and externally was also weak and may be a reason why collaboration with other agencies was not in focus. Communication network was overloaded quickly. The process of paging different physicians did not work during the exercise. Overloaded telephone network and other problems with IP telephony have been emphasized in many studies. The former was recently reported in the terrorist attack in Brussels [2, 13, 25].

The main goal for evaluation of ED was to assess the organizational structure and not the medical assessment of the victims. All patients were medically assessed by an experienced surgical team at trauma bay and based on ATLS guidelines. It is, however, important to emphasize that exercises with no real-time treatment schedule, with or without figurants or patient cards, cannot generate measurable parameters and thus are not fully evaluable.

## Limitations

In this study, an observational method was used to study the exercise [15–17]. The main advantage of observing exercises is twofolds: (1) the possibility of following the event directly as it happens and (2) the documentation of the participant’s behavior from the arrival of the first unit until the situation is normalized and the exercise has ended [18–21]. This method is more favorable than a retrospective study in which post-event data collection can be difficult and the events can be viewed as vague reconstructions. In addition, the participants often have difficulty remembering exactly what happened, in what order things were done, and who did what [23, 28, 29]. The limitation with this method is, however, the observer himself, since the evaluation of the exercise is based on evaluator’s background, experience, and his/her ability to picture the whole working process without any involvement and in a passive stance. Using free text as comment often express an individual perception and may also be individually comprehended.

Although we used VAS scale to measure some parameters, the points given are also individual-based. Some studies recommend measurable parameters and performance indicators such as time consumed with different actions [27]. However, a quick decision does not necessarily equal the right and best solution.

A better evaluation would be possible if the whole chain of actions, including prehospital care, triage, transport to the ED and from ED to the ward, could be involved. This would probably show even weaknesses at the prehospital level with impacts on the decision made at the hospital.

## Conclusion

In conclusion, the immediate response to a major incident such as a terror attack is a combination of medical and non-medical measures in a multidisciplinary manner. Although quick and appropriate medical intervention is vital for the survival of victims, the non-medical measures, such as establishing command and control, safety, proper communication, collaboration with other agencies, information, logistic, etc, are equally important. Any kind of multidisciplinary approach, including CM collaboration, should be harmonized by mutual planning, training, and exercising.
